# TLR1/2 orchestrate human plasmacytoid predendritic cell response to gram+ bacteria

**DOI:** 10.1371/journal.pbio.3000209

**Published:** 2019-04-24

**Authors:** Salvatore Raieli, Coline Trichot, Sarantis Korniotis, Lucia Pattarini, Vassili Soumelis

**Affiliations:** 1 Institut Curie, Centre de Recherche, PSL Research University, Paris, France; 2 INSERM U932, Immunity and Cancer, Paris, France; New York University School of Medicine, UNITED STATES

## Abstract

Gram+ infections are worldwide life-threatening diseases in which the pathological role of type I interferon (IFN) has been highlighted. Plasmacytoid predendritic cells (pDCs) produce high amounts of type I IFN following viral sensing. Despite studies suggesting that pDCs respond to bacteria, the mechanisms underlying bacterial sensing in pDCs are unknown. We show here that human primary pDCs express toll-like receptor 1 (TLR1) and 2 (TLR2) and respond to bacterial lipoproteins. We demonstrated that pDCs differentially respond to gram+ bacteria through the TLR1/2 pathway. Notably, up-regulation of costimulatory molecules and pro-inflammatory cytokines was TLR1 dependent, whereas type I IFN secretion was TLR2 dependent. Mechanistically, we demonstrated that these differences relied on diverse signaling pathways activated by TLR1/2. MAPK and NF-κB pathways were engaged by TLR1, whereas the Phosphoinositide 3-kinase (PI3K) pathway was activated by TLR2. This dichotomy was reflected in a different role of TLR2 and TLR1 in pDC priming of naïve cluster of differentiation 4^+^ (CD4^+^) T cells, and T helper (Th) cell differentiation. This work provides the rationale to explore and target pDCs in bacterial infection.

## Abstract 2

It is unclear whether and how plasmacytoid pre-dendritic cells (pDCs) respond to bacteria; this study shows that human pDCs sense Gram-positive bacteria through the Toll-like receptors TLR1 and TLR2, and that these receptors have distinct roles in the activation of pDCs.

## Introduction

Tuberculosis (TB) and multidrug-resistant bacteria are a major concern for worldwide health [1]. In TB and gram+ infection, type I interferon (IFN) has been shown to play a pathological role [2,3]. Plasmacytoid pre-dendritic cells (pDCs) are known to produce high amounts of type I IFN in response to viral sensing [4]. It is reported that pDCs are able to respond to gram+ bacteria [5], can be recruited at the site of the infection, and are enriched in TB lymph nodes [6].

Gram+ bacteria express lipoproteins on their surface membrane, which play an important role in their survival and pathogenicity [7]. Bacterial lipoproteins are recognized by toll-like receptor (TLR)1/2 and induce activation and maturation in dendritic cells (DCs) [8]. TLR2 knockout mice are more susceptible to mycobacterial infection, but *Mycobacterium tuberculosis* is able to hijack TLR2 signaling to enhance its survival in the host [9]. TLR1 and TLR2 expression in human pDCs has not been reported [10], leading to the conclusion that TLR 1 and 2 do not have a functional role in pDCs.

Human pDCs express mainly TLR7 and TLR9, localized in the endosomes and capable of sensing nucleic acids [10–12]. pDCs also express a range of cytosolic sensors, either at steady state, such as the helicases DEAH box protein 9 (DHX9) and DHX36 [13], or following innate activation, such as retinoic acid inducible gene 1 (RIG-I) [14]. However, how pDCs sense gram+ bacteria is still debated, and their role in gram+ infections is still poorly investigated [15].

Here, using human primary cells, we provide definite evidence that pDCs sense gram+ bacteria through TLR1 and TLR2.

## Results

### Human pDCs respond to bacterial lipoproteins through TLR1 and TLR2

In order to investigate how pDCs sense gram+ bacteria, we screened steady-state blood pDCs for TLR mRNA expression. In addition to the known expression of TLR7 and TLR9, we detected low levels of TLR1, TLR2, TLR6, and TLR10 (S1A Fig). Among the TLRs expressed by pDCs, TLR1 and TLR2 mediate bacterial sensing by binding lipoproteins [8].

**Fig 1:**
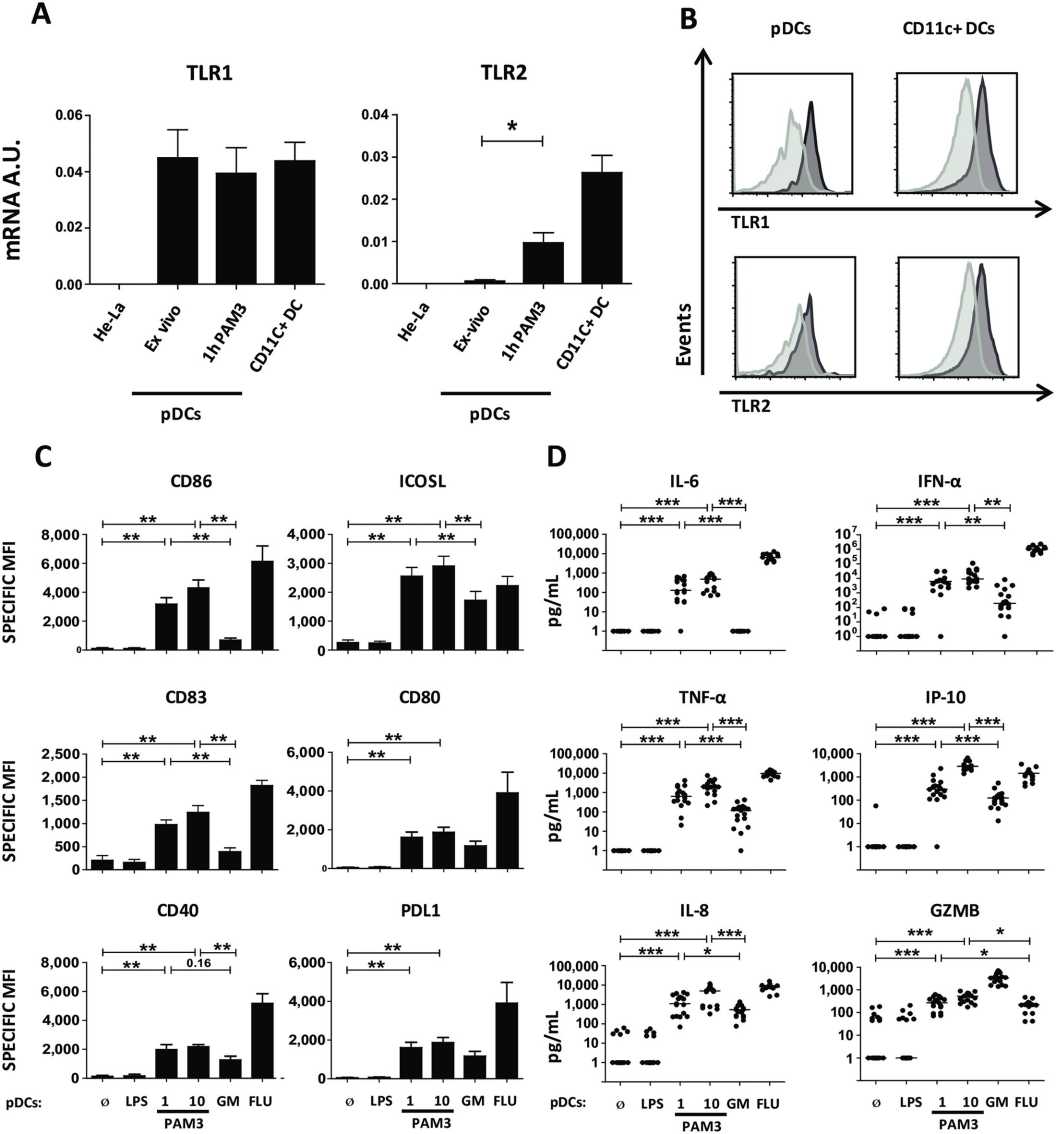
Human pDCs express TLR1/2 and respond to PAM3. (A) RT-PCR quantification of TLR1 and TLR2 expression from total mRNA of sorted human blood pDCs before and after 1-hour activation with PAM3 as compared to CD11c^+^ DCs and HeLa cells. Results were normalized on 3 housekeeping genes. Results include 5 donors. (B) pDCs and CD11c^+^ DCs were stained in freshly isolated PBMCs with anti-TLR1 and anti-TLR2 antibody (dark gray), respective cognate isotype (light gray). (C–D) Sorted human pDCs were cultured during 24 hours with medium (Ø), 0.1 μg/mL LPS, 1 and 10 μg/mL PAM3, 100 ng/mL GM, or 82 HA/ml FLU. (C) Specific MFI for surface expression of costimulatory or coinhibitory molecules from activated pDCs by FACS. Results include the mean of 9 donors. (D) Cytokine secretion by pDCs. Results include the mean of 17 donors. Each dot represents a donor. *p < 0.05; **p < 0.01; ***p < 0.001 (Wilcoxon test). Underlying data for this figure can be found in S1 Data. AU, arbritrary unit; CD, cluster of differentiation; DC, dendritric cell; FACS, fluorescence-activated cell sorting; FLU, influenza virus; GM, GM-CSF; Gm-CSF, granulocyte-macrophage colony-stimulating factor; GZMB, Granzyme B; ICOSL, inducible T cell costimulator ligand; HA, hemagglutinin; IFN, interferon; IL, interleukin; IP-10, Interferon gamma-induced protein 10; LPS, lipopolysaccharide; PBMC, peripheral blood mononuclear cell; PAM3, PAM3CSK4; pDC, Plasmacytoid predendritric cell; PDL1, programmed cell death ligand 1; RT, real time; TLR, toll-like receptor; TNF, tumor necrosis factor.

We measured pDC TLR1 and TLR2 mRNA expression on freshly isolated blood pDC and following stimulation with PAM3CSK4 (PAM3), a bacterial lipoprotein used as a prototypical TLR1/2 ligand. HeLa cells were used as negative control and CD11c^+^ DCs as positive control for the expression of TLR1/2. pDCs maintained a stable TLR1 mRNA expression following stimulation. PAM3 activation increased TLR2 expression as compared with ex vivo pDCs (Fig 1A).

We further investigated whether pDCs express TLR1 and TLR2 at the protein level. Using flow cytometry, we confirmed in freshly isolated peripheral blood mononuclear cells (PBMCs) that pDCs expressed TLR1 and TLR2 at their surface, as compared to isotype control (Fig 1B and quantification in S1B Fig).

To address the functionality of TLR1 and 2 on pDC, we investigated pDC response to PAM3 after 24 hours of stimulation. We observed up-regulation of costimulatory molecules (CD86, inducible T cell costimulator ligand (ICOSL), CD83, CD80, CD40 and programmed cell death ligand 1 (PDL1)) and MHC-II expression (HLA-DR) on the surface (Fig 1C, S1C and S1D Fig), as compared to untreated pDCs and lipopolysaccharide (LPS)-treated pDCs. As expected, pDCs activated by influenza virus (FLU) or granulocyte-macrophage colony-stimulating factor (GM-CSF) expressed CD86, ICOSL, CD83, CD80, CD40, and PDL1 (Fig 1C) [16]. pDCs stimulation with PAM3 induced a higher CD40, CD86, ICOSL, and CD83 expression in comparison with GM-CSF. As expected, FLU induced a stronger expression of checkpoints compared with both PAM3 and GM-CSF (Fig 1C).

A feature of pDCs is high type I IFN secretion. The ability of PAM3 to induce type I IFN secretion in human pDCs has been questioned [17,18]. Here, highly pure (99%) pDCs responded to 1 and 10 μg/ml of PAM3 by secreting type I IFN (Fig 1D). In addition, PAM3 induced the secretion of pro-inflammatory cytokines (interleukin [IL]-6, tumor necrosis factor [TNF]-α), and chemokines (IL-8, IP-10), although to a lower extent than with FLU (Fig 1D). Furthermore, pDCs secreted Granzyme B (GZMB) in response to bacterial lipoprotein stimulation (Fig 1D). Both 1 and 10 μg/ml of PAM3-induced pDCs the expression of costimulatory molecules and cytokine secretion at comparable levels (Fig 1C and 1D).

We used PAM3 to stimulate pDCs purified from tonsils, a site of frequent encounter with gram+ bacteria. Tonsillar pDCs up-regulated surface costimulatory molecules (CD86, CD80, CD40, and PDL1) (S1E Fig) and MHC-II complex in line with our data on blood pDCs (S1D Fig).

These data suggest that pDCs from both blood and from physiological bacterial interfaces functionally respond to bacterial lipoproteins.

### TLR1/2 pathway is necessary for pDC response to gram+ bacteria

**Fig 2:**
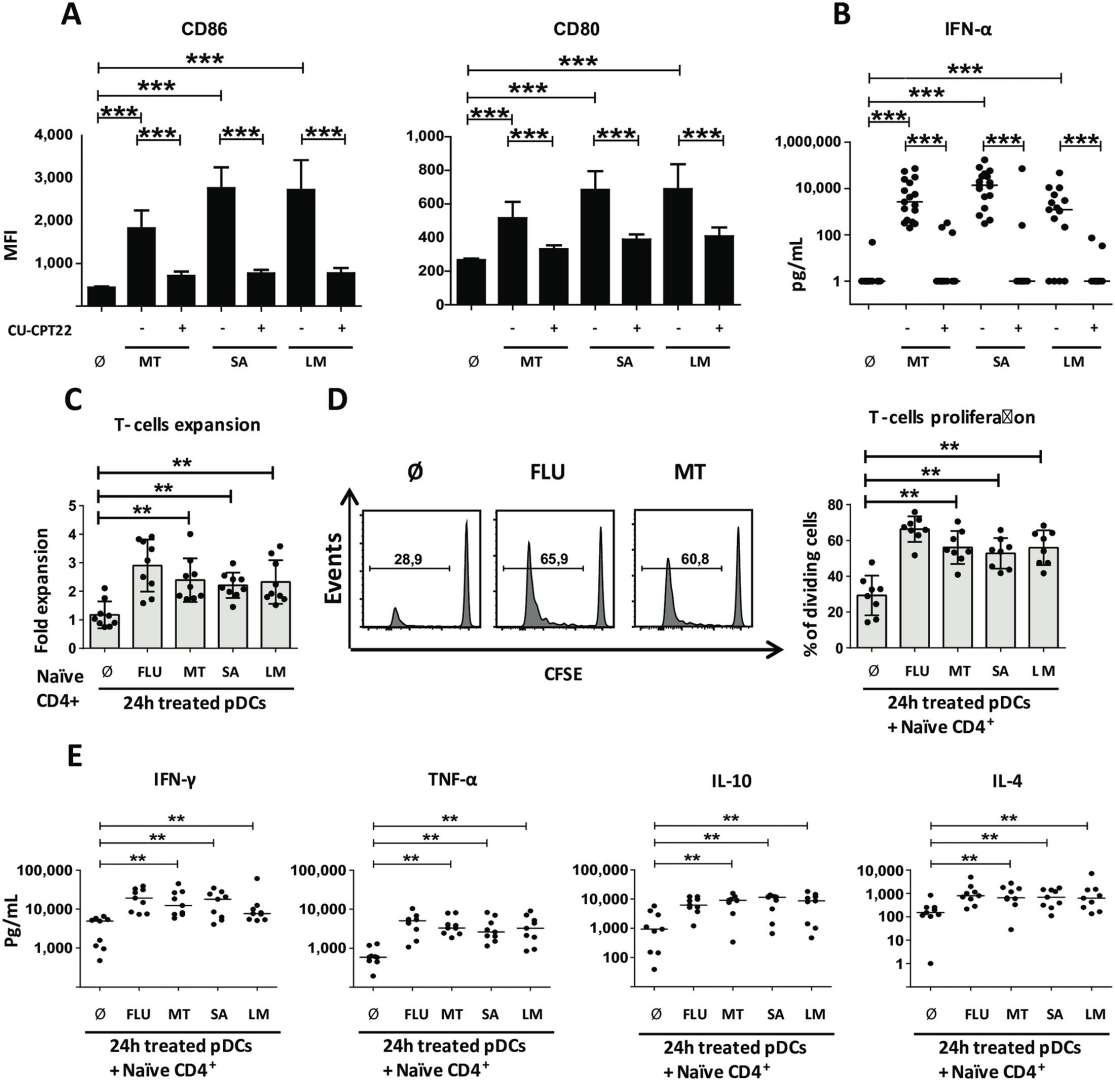
pDCs sense different gram+ bacteria through TLR1/2 pathway. (A–B) Sorted human blood pDCs were cultured during 24 hours with medium either without (Ø) or with: heat-killed MT, heat-killed SA, or heat-killed LM and in presence (+) or absence (−) of CU-CPT22. (A) MFI for surface expression of costimulatory molecules from activated pDCs. Results include the mean of 27 independent donors. (B) Cytokine secretion by pDCs. Results include 17 independent donors. (C–D) Allogeneic CD4^+^ naive expansion and percentage of dividing living cells after 6 days of coculture with 24-hours gram^+^-stimulated pDCs. FLU activated pDCs were used as a control. Results include the mean of 9 donors. (E) Th cytokine pattern from gram+ pDCs activated T-cell coculture. Cytokines were measured after 24 hours polyclonal restimulation of the T cells. Results include the mean of 9 independent donors. Each dot represents a donor. *p < 0.05; **p < 0.01; ***p < 0.001 (Wilcoxon test). Underlying data for this figure can be found in S1 Data. CD, cluster of differentiation; FLU, influenza virus; LM, *Listeria monocytogenes*; MT, *Mycobacterium tuberculosis*; pDC, Plasmacytoid predendritric cell; SA, *Staphylococcus aureus*; Th, T helper; TLR, toll-like receptor.

We next questioned whether, in addition to purified lipoproteins, pDCs could respond to whole gram+ bacteria. Although pDC activation by *Staphylococcus aureus* was reported [5], whether pDCs can respond to *M*. *tuberculosis* is still debated [6]. Sorted blood pDCs were stimulated with 3 different heat-killed gram+ bacteria relevant to human infections: *M*. *tuberculosis*, *S*. *aureus*, and *Listeria monocytogenes*. We observed up-regulation of CD80 and CD86 following pDC culture with heat-killed bacteria (Fig 2A). To establish the role of TLR1/2, we took advantage of a chemical antagonist for both TLR1 and 2, CU-CPT22 [19]. CU-CPT22 did not affect unstimulated pDCs, nor did it impact costimulatory molecule expression (CD80, CD86) or type I IFN secretion in FLU-activated pDCs (S2A–S2C Fig). On the contrary, CU-CPT22 treatment strongly decreased bacteria-induced CD80 and CD86 expression by pDCs (Fig 2A and S2D Fig). Furthermore, gram+-stimulated pDCs secrete high amount of type I IFN (Fig 2B) thus indicating full activation of pDCs by bacteria (Fig 2B). TLR1/2 blocking by CU-CPT22 almost completely abrogated type I IFN production (Fig 2B). Therefore, pDCs responded to whole gram+ bacteria in a TLR1/2-dependent manner.

T-cell priming is an important adaptive function of activated pDCs [20]. We investigated whether gram+-stimulated pDCs control T-cell priming. pDCs primed with gram+ bacteria, or FLU as a positive control, were cultured with allogeneic naive CD4^+^ T cells for 6 days. Bacteria-primed pDCs induced CD4^+^ T-cell expansion (Fig 2C) and proliferation (Fig 2D) comparable to FLU-activated pDCs (Fig 2C and 2D). After 6 days of coculture, T cells were polyclonally restimulated to measure T helper (Th) cytokine production. Gram+ bacteria–activated pDCs induced secretion of IL-4, IFN-γ, and TNF-α from CD4 T cells (Fig 2E). Additionally, we detected IL-10 (Fig 2E). Overall, these cytokines suggest a diversity of Th cell cytokine patterns induced by bacterial-activated pDCs: Th1 (IFN-γ), Th2 (IL-4), and T regulatory (Treg) (IL-10).

CD11c^+^ DCs are known to express TLR1/2 and to be able to induce Th cell differentiation. We investigated the differences in naïve CD4^+^ T-cell priming by PAM3-activated CD11c^+^ DCs and pDCs (S2E Fig). T cells primed with PAM3-activated CD11c^+^ DC or pDCs showed a comparable state of activation. However, pDCs induced a prominent Th2-like profile compared with CD11c^+^ DCs (higher secretion of IL-4, IL-5, and IL-10), suggesting different contributions to immune regulation in the context of bacterial infection (S2E Fig).

To establish whether TLR1/2-activated pDCs were able to induce cytokine production by memory T cells, we cultured PAM3-activated pDCs with allogeneic memory CD4^+^ T cells from healthy donor peripheral blood. Memory CD4^+^ T cells secreted significant amounts of IFN-γ, IL-10, IL-3, IL-4, and IL-9 when cocultured with PAM3-activated pDCs compared with memory CD4 T cells cocultured with untreated pDCs (S3A Fig). The amounts of these cytokines were comparable to FLU condition and much higher than the negative control LPS. Moreover, PAM3-activated pDCs were the only ones capable of inducing the production of both IL-17A and IL-17F from memory CD4^+^ T cells as compared with untreated pDCs and FLU-pDCs. This shows that PAM3-activated pDCs are capable of inducing effector cytokine production by memory CD4^+^ T cells, including IL-17A and F, important in epithelial immunity.

Recent results demonstrated the existence of a rare DC subset defined as DC5 or AXL^+^SIGLEC6^+^ (AS-DC) [21]. This subset is characterized by the expression of the surface markers CD2, CD5, and AXL receptor tyrosine kinase (AXL) but also shares some markers with pDCs, leading to potential contamination of the pDC population. In order to determine whether pure pDCs (DC5-depleted pDCs) were able to induce T-cell expansion and Th polarization to the same extent as LIN^-^CD4^+^CD11c^-^ pDCs, we cell sorted pure pDCs following the presented gating strategy (S3B Fig). CD2^-^CD5^-^AXL^-^ pDCs were activated for 24-hours with PAM3, FLU, LPS, or GM-CSF and cocultured with allogeneic naïve CD4^+^ T cells from healthy peripheral blood. We found that TLR1-activated pure pDCs were capable of inducing CD4 T-cell expansion and Th cell differentiation (S3C Fig), with increased production of IFN-γ, IL-10, IL-3, IL-4, IL-9, and GM-CSF as compared with nontreated pDCs. These results show that CD4^+^ T-cell expansion and Th cell differentiation induced by TLR1-activated pDCs is not due to contamination with DC5.

### TLR1 and TLR2 play a differential role in the pDCs response to bacterial lipoproteins

**Fig 3:**
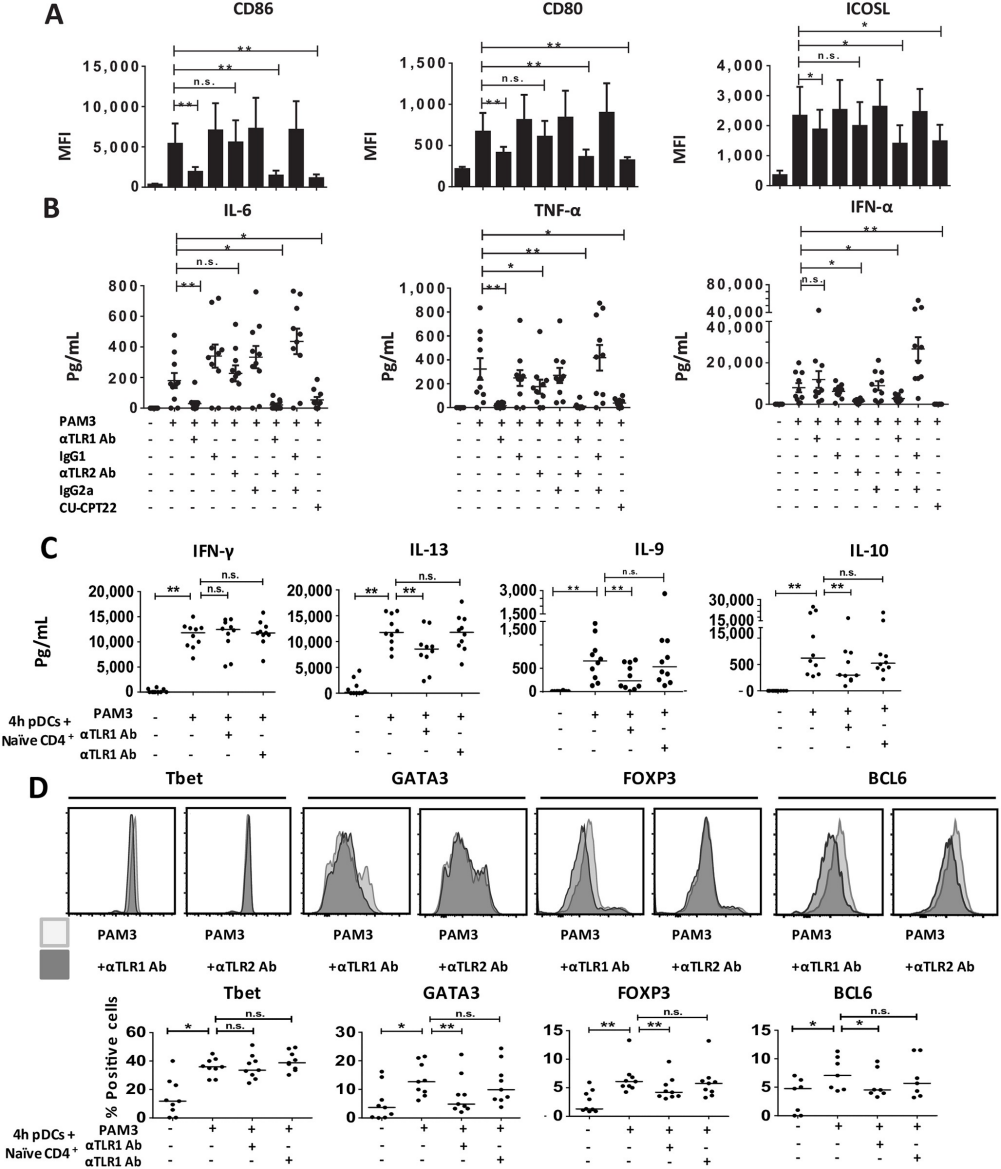
TLR1 and TLR2 functional blocking has a differential impact on pDC innate and adaptive functions. (A–B) Sorted human pDCs were cultured during 24 hours with medium (Ø) or PAM3 in the presence or not of TLR1 neutralizing antibody (αTLR1 Ab), TLR2 neutralizing antibody (αTLR2 Ab), or IgG1 isotype control antibody (IgG1); or IgA2 Isotype Control (IgG2a), double blocking (TLR1 Ab + αTLR2 Ab), double control isotype (IgG1 + IgG2a), or CU-CPT22. (A) MFI for surface expression of costimulatory molecules. Results include the mean of 9 independent donors. (B) Cytokine secretion by pDCs. Results include the mean of 10 independent donors. Each dot is an independent donor. (C–D) The 24-hour–stimulated pDCs were cocultured with allogeneic CD4^+^ naive T cells during 6 days. (C) Th cytokine quantification. Cytokines were measured after 24-hour polyclonal restimulation of the T cells. Each dot is an independent donor, *n* = 10. (D) Percentage of Th master regulator expression. Intracellular FACS was performed after 4 days of coculture. Results include the mean of 9 independent donors for Tbet, GATA3, and FOXP3. Results show the mean of 7 independent donors for BCL-6. Each dot represents a donor. *p < 0.05; **p < 0.01; ***p < 0.001 (Wilcoxon test). Underlying data for this figure can be found in S1 Data. Ab, antibody; BCL-6, B-cell lymphoma 6; CD, cluster of differentiation; CU-CPT22,; FACS, fluorescence-activated cell sorting; FOXP3, forkhead box P3; GATA3, GATA binding protein 3; ICOSL, inducible T cell costimulator ligand; IFN, interferon; IgG, Immunoglobulin G; IL, interleukin; MFI, mean fluorescence intensity; n.s., not significant; PAM3, PAM3CSK4; pDC, Plasmacytoid pre-dendritric cell; Tbet, T-box transcription factor TBX21; Th, T helper; TLR, toll-like receptor; TNF, tumor necrosis factor.

In order to investigate the differential contribution of TLR1 and TLR2 in mediating pDC response to bacterial lipoproteins, we separately blocked the 2 receptors with specific antibodies, as compared with matched isotype controls [22,23]. TLR1 functional blocking significantly reduced costimulatory molecule expression (CD80, CD86, and ICOSL), whereas TLR2 blockade did not (Fig 3A and S4A Fig). TLR1 blocking almost completely abolished secretion of the pro-inflammatory cytokines IL-6 and TNF-α (Fig 3B). Conversely, TLR2 blocking inhibited type I IFN secretion, which was not impacted by TLR1 blocking (Fig 3B). Combined TLR1 and TLR2 blockade, as well as the TLR1/2 competitive antagonist CU-CPT22, inhibited both costimulatory molecule expression and cytokine release (Fig 3A and 3B). These results suggest a differential control of pDC functions by TLR1 and TLR2.

Next, we performed coculture experiments with PAM3-treated pDCs and naive CD4^+^ T cells, with and without TLR1 or TLR2 blocking antibodies. TLR1 blocking during PAM3 activation reduced T-cell expansion and proliferation (S4B and S4C Fig). Following polyclonal restimulation, we did not detect any difference in the Th1 prototypical cytokine IFN-γ (Fig 3C and S4E Fig). However, TLR1 blocking in pDCs decreased prototypical Th2 cytokines (IL-13, IL-4, IL-5) (Fig 3C and S4E Fig). TLR1 blocking also diminished IL-10 production by Th cells, suggesting a decrease in Treg generation (Fig 3C). We found that TLR1 blocking reduced IL-9 secretion by Th cells (Fig 3C). After 4 days of pDCs-T cell coculture, we performed intracellular staining for Th master regulator transcription factors to better characterize the Th subsets induced. TLR1/TLR2 blocking did not reduce Tbet induction (Fig 3D and S4D Fig), in line with our observation on IFN-γ production. However, TLR1 blocking diminished GATA3 and FOXP3 expression (Fig 3D and S4D Fig), in line with its impact on Th2 and Treg polarization. TLR1 blocking also reduced BCL-6 expression (Fig 3D and S4D Fig), involved in T-follicular helper (Tfh) generation [24].

### TLR1 and TLR2 activate different signaling pathways in response to bacterial lipoproteins

**Fig 4:**
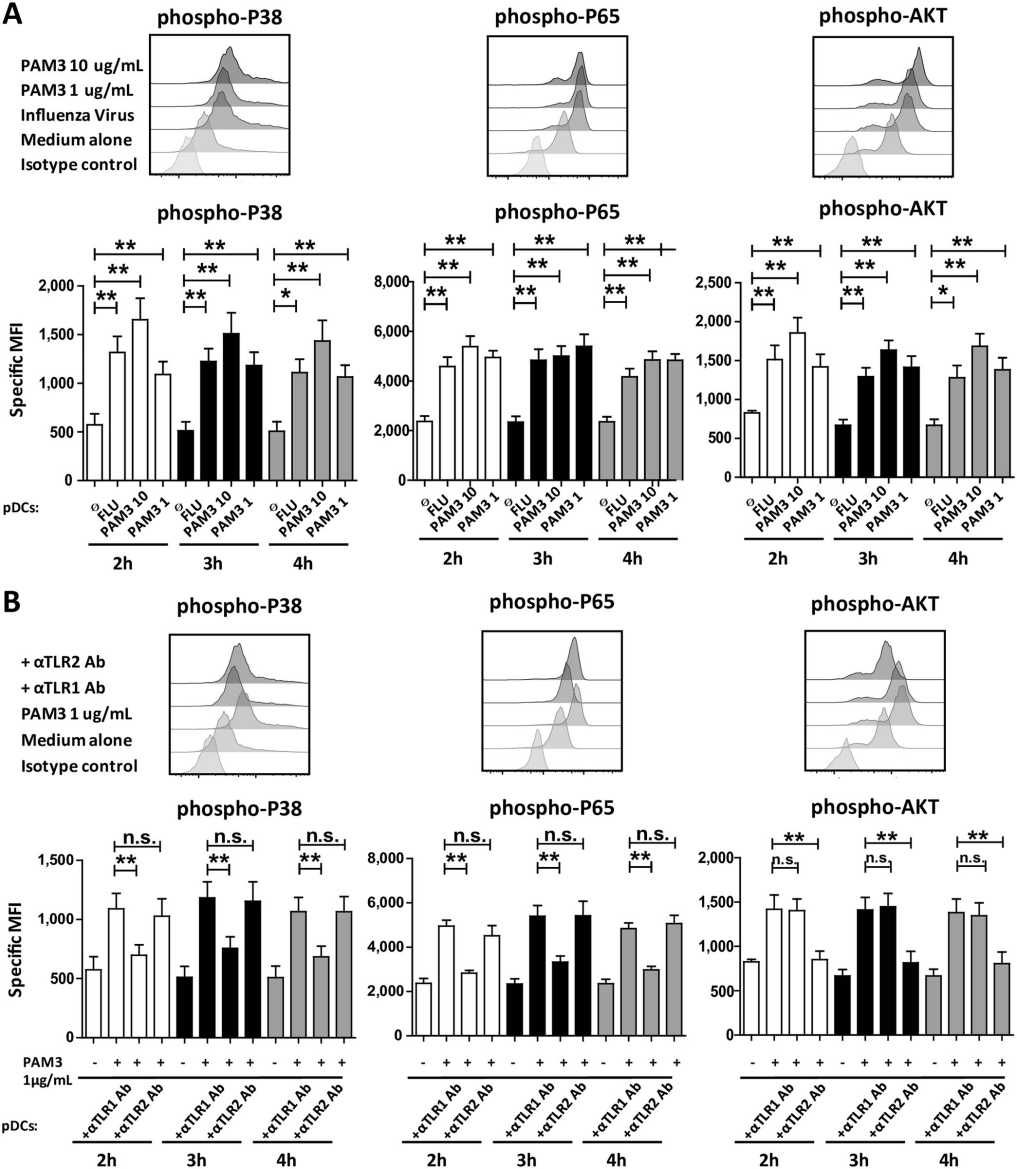
TLR1 and TLR2 exploit distinct pathways following PAM3 stimulation. (A–B) Sorted human blood pDCs were cultured during 4-hours with only medium (Ø) and with or without PAM3 in the presence or not of TLR1 neutralizing antibody (αTLR1 Ab), TLR2 neutralizing antibody (αTLR2 Ab), IgG1. FLU was used as control. (A–B) p38 MAPK (first panel), p65 NF-κB (second panel), AKT PI3K (third panel) at 3-hours. p38 MAPK (first panel), p65 NF-κB (second panel), AKT PI3K (third panel) at 3 different time points (2, 3, and 4 hours). Results include the mean of 8 independent donors. *p < 0.05; **p < 0.01; ***p < 0.001 (Wilcoxon test). Underlying data for this figure can be found in S1 Data. Ab, antibody; AKT, AKT serine/threonine kinase; FLU, influenza virus; IgG, Immunoglobulin G; MAPK, Mitogen-activated protein kinases; MFI, mean fluorescence intensity; NF-κB, nuclear factor kappa-light-chain-enhancer of activated B cells; PAM3, PAM3CSK4; pDC, Plasmacytoid predendritric cell; PI3K, Phosphoinositide 3-kinase; TLR, toll-like receptor.

In pDCs, MAPK and NF-κB pathway activation leads to costimulatory expression and pro-inflammatory cytokine release, whereas PI3K signaling controls Type I IFN induction [25]. In the case of TLR7 and TLR9, these 2 signaling pathways are activated in early and late endosomes, respectively [26]. We performed phospho-fluorescence-activated cell sorting (phosphoFACS) to investigate which pathways were activated by bacterial lipoproteins in pDCs. Stimulation with PAM3 (1 and 10 μg/mL) led to p38, p65, and AKT serine/threonine kinase AKT phosphorylation as compared with untreated pDCs (Fig 4A). pDC stimulation with FLU virus was used as positive control (Fig 4A). These results suggested that MAPK, NF-κB, and PI3K were activated following bacterial lipoproteins activation.

Next, we tested how TLR1/2 blocking affected intracellular signaling cascades. TLR1, but not TLR2, blocking reduced p38 and p65 phosphorylation in pDCs activated with PAM3 (Fig 4B). On the contrary, TLR2 blocking diminished AKT phosphorylation in comparison with PAM3-treated pDCs whereas TLR1 blocking did not show an effect (Fig 4B). We observed this inhibition after 2, 3, and 4-hours of PAM3 activation (Fig 4B).

These data suggest that the mechanism behind the differences observed in pDCs innate versus adaptive responses following TLR1 and TLR2 blocking is related to different signaling pathways controlled by the 2 receptors.

## Discussion

pDCs are known to express a narrow TLR pattern that is restricted to TLR7 and TLR9 [10]. Accordingly, TLR1 and TLR2 expression was considered a prototypical feature of myeloid cells and absent from pDCs [10]. The low expression level of TLR1/2 on pDCs as compared with TLR7 and 9 may have previously suggested that it is not functionally relevant. However, peripheral blood pDCs are considered the major source of type I IFN following *S*. *aureus* stimulation [15]. We found that pDCs express TLR1 at steady state and TLR2 in a stimulation-dependent manner, and that those 2 TLRs are functional for PAM3 sensing.

Commensal bacteria have an immunomodulatory impact in the gut. Some of them, such as *Bacteroides fragilis* and Clostridia, are gram+ [27,28]. Here, we show that pDCs respond to the lipoprotein characteristic of gram+ bacteria and that lipoprotein-activated pDCs induced IL-10 and FOXP3 expression in CD4^+^ T cells. pDCs are present in the human gut at steady state [29]. However, other groups report that pDCs can participate in sustaining inflammation in acute colitis [30]. Our study suggests that pDCs, following bacterial sensing, could instruct CD4^+^ T cells in the gut and promote a mixed Th cell cytokine profile—including a regulatory phenotype—but also cytokines prototypical of Th1, Th2, and Th17 inflammation. Therefore, a detailed investigation of pDC role in the gut is warranted. Our results provide a strong basis for a functional link between pDCs and gram+ bacteria in various physiopathological contexts.

Our data show that GZMB can be induced by bacterial lipoproteins. It has been shown that pDCs in TB patients’ lymph nodes produce GZMB [6]. Our data suggest that bacterial sensing through TLR1/2 could induce GZMB in pathological conditions, such as TB infection.

It has been proposed that bacterial nucleic acids can activate pDCs through such intracellular sensors as TLR7 and TLR9, but this requires phagocytosis [15]. However, pDCs are poorly phagocytic cells [5], suggesting the possible implication of putative extracellular sensors. Our results provide the first evidence that TLR1/2 surface receptors are necessary for pDC response to gram+ bacteria.

In TB, a diversity of Th responses has been observed [31]. It has been proposed that Th1 is the protective response in TB and in many gram+ bacterial infections, whereas Th2 and Treg have been shown to promote the disease [31–34]. In atopic dermatitis, in which there is a strong link between disease flare and *S*. *aureus* skin infection [35], it has been shown that both Th1 and Th2 responses coexist [36]. In vitro, we showed that gram+ stimulation of pDCs induced a mixed Th1, Th2, and Treg cytokine profile, suggesting that they could contribute to the in vivo–observed Th diversity.

Our results showed that TLR1 and TLR2 play a different and complimentary role in the pDC response to bacterial lipoproteins. Although it is reported that TLR2 in inflammatory monocytes can be endocytosed and activate IRF7 in response to a viral ligand [37], our results are the first to link a type I IFN response and the TLR2 pathway in response to a bacterial ligand.

Furthermore, we observed that TLR1 and TLR2 blocking on pDCs had differential effects on Th cytokine secretion. TLR1 blocking on pDCs decreased T-cell polarization toward Th2, Treg, and Tfh but not Th1 cells. Conversely, TLR2 blocking showed a specific inhibition of Type I IFN secretion without impacting T-cell polarization. These data show that TLR1 activation could promote an adaptive response (costimulatory molecule expression, pro-inflammatory cytokine secretion, Th proliferation and polarization), whereas TLR2 activation induced type I IFN, which broadly functions in innate immunity. Our data suggest that the innate and/or adaptive response of pDCs could be differentially targeted.

These data suggest that the mechanism behind the differences observed in pDCs’ innate versus adaptive responses following TLR1 and TLR2 blocking is related to different signaling pathways controlled by the 2 receptors.

Our findings open broad perspectives on the possible role of pDCs in gram+ bacterial diseases. Here, we showed that *M*. *tuberculosis*, *S*. *aureus*, and *L*. *monocytogenes* induced high levels of type I IFN production by pDC and that this is abrogated by TLR1/2 antagonist (CU-CPT22). Type I IFN is highly expressed in TB, in which it has been proposed to dampen immune response [38]. Therefore, our data establish pDCs as a possible source of type I IFN in TB-infected tissues. Furthermore, TLR1 polymorphisms are associated with TB susceptibility [39,40]. Future studies are required to establish whether pDCs could represent a pharmacological target in TB. In the past few years, different attempts to develop a vaccine direct against of *S*. *aureus* have failed [41]. Subsequently, lipoproteins have been considered promising candidates [7]. Besides, vaccines in combination with TLR7 ligand show a boost in the protective immunity [42]. Our results suggest the possible role of pDCs in vaccine efficacy considering their capacity to respond to lipoproteins, high TLR7 expression, and capacity to prime T cells in response to gram+ bacteria.

## Materials and methods

### Ethic statement

Blood buffy coats from healthy donors were obtained from the French blood bank (Etablissement Français du Sang) through an approved convention (N° 18/EFS/033). Tonsils from patients undergoing tonsillectomy for obstructive sleep apnea were obtained from Hôpital Necker (Paris, France) as surgical residues, according to the French legislation (public health law, art L 1121-1-1, art L 1121-1-2).

### Blood samples and cell isolation

PBMCs were isolated by Ficoll density gradient centrifugation (Ficoll-Paque, GE Healthcare, Chicago, IL). pDCs and CD11c^+^ DCs were isolated by a first step of total DC enrichment (EasySep human Pan-DC Enrichment kit, Stemcell, Canada) followed by FACS sorting as Lineage^−^CD11c^−^CD4^+^ to a 99% purity [20]. Tonsil pDCs were isolated using the following protocol by Durand and Segura [43]. DC5^-^ pDCs was isolated by a first step of total DC enrichment (EasySep human Pan-DC Enrichment kit, Stemcell, Canada) followed by FACS sorting as Lineage^−^CD11c^−^CD4^+^CD2^−^CD5^−^AXL^−^ to 99% purity. Human naive CD4^+^ T cells were isolated from PBMCs by negative selection (naïve CD4 T-cell isolation kit, Miltenyi, Germany) to a >98% purity. Total Memory CD4^+^ T cells were isolated from PBMCs by negative selection (Memory CD4^+^ T Cell isolation Kit and LS columns, Miltenyi, Germany).

### Flow cytometry

PMBCs were stained with FITC anti-CD3 (BD, Franklin Lakes, NJ), FITC anti-CD14 (BD, Franklin Lakes, NJ), FITC anti-CD16 (BD, Franklin Lakes, NJ), FITC anti-CD19 (Miltenyi, Germany), PECy7 anti-CD11c (BD, Franklin Lakes, NJ), VioGreen anti-CD4 (Miltenyi, Germany), PE anti-TLR1 (eBioscience (Thermo Fisher Scientific), Waltham, Ma), and AlexaFluor700 anti-TLR2 (eBioscience (Thermo Fisher Scientific), Waltham, Ma). After culture, cells were stained with 4,6-diamidino-2-phenylindole (DAPI; Sigma-Aldrich, Saint Louis, MO) that was added before acquisition to exclude dead cells. pDCs were stained with the following antibodies: AF700 anti-HLA-DR (Biolegend, San Diego, Ca), APC anti-ICOSL (R&D, Minneapolis, MN), PE anti-CD86 (BD, Franklin Lakes, NJ), FITC anti-CD80 (BD, Franklin Lakes, NJ), FITC anti-CD40 (BD, Franklin Lakes, NJ), Percp5.5 anti-CD83 (eBioscience (Thermo Fisher Scientific), Waltham, Ma), and Percp5.5 anti PD-L1 (eBioscience (Thermo Fisher Scientific), Waltham, Ma). Tonsil pDCs were stained with the following antibodies: isotype-matched antibodies Percp5.5 anti PD-L1 (eBioscience (Thermo Fisher Scientific), Waltham, Ma), PE anti-CD80 (BD, Franklin Lakes, NJ), FITC anti-CD40 (BD, Franklin Lakes, NJ), Brillant violet 650 anti-CD86 (Biolegend, San Diego, Ca), and AF780 anti-HLA-DR (eBioscience (Thermo Fisher Scientific), Waltham, Ma). For intracellular staining, CD4 naive T cells were cultured for 4 days with allogeneic activated pDCs (PAM3 in combination with anti-TLRs antibody). T cells were stained with ZombieNir fixable kit (Biolegend, San Diego, Ca) before surface staining, fixation, and permeabilization (FOXP3 Fix/Perm buffers; eBioscience (Thermo Fisher Scientific), Waltham, Ma). Cells were then stained with APC anti BCL-6 (BD, Franklin Lakes, NJ), PercP55 anti Tbet (BD, Franklin Lakes, NJ), Pecy7 anti GATA3 (eBioscience (Thermo Fisher Scientific), Waltham, Ma), and APC anti FoxP3 (eBioscience (Thermo Fisher Scientific), Waltham, Ma). Isotype-matched antibodies were used as control. For phosphoFAC*S*, pDCs were treated for 4 hours with medium, PAM3 (in combination with neutralizing antibody as described before), and FLU. Cells were fixed with Fix Buffer I (BD, Franklin Lakes, NJ) and permeabilized with Perm Buffer III (BD, Franklin Lakes, NJ). Cells were stained with PE anti-p-AKT (BD, Franklin Lakes, NJ), PECy7 anti-p-p65 (BD, Franklin Lakes, NJ), and PE anti-p-p38 (Cell signaling, Danvers, Ma). Isotype-matched antibodies were used as control. Cells were analyzed on a flow cytometer (blood pDCs on BD LSRII, tonsil pDCs and T cells on BD Fortessa), and data were processed using FlowJo software (FlowJo LLC, Ashland, OR.).

### pDC culture

pDCs were cultured in RPMI 1640 Medium, GlutaMAX (Life Technologies (Thermo Fisher Scientific), Waltham, Ma) containing 10% Fetal Calf Serum (Hyclone (Thermo Fisher Scientific), Waltham, Ma), 100 U/ml Penicillin/Streptomycin (GIBCO (Thermo Fisher Scientific), Waltham, Ma), MEM Non Essential Amino Acids (GIBCO (Thermo Fisher Scientific), Waltham, Ma), and 1mM NA pyruvate (GIBCO (Thermo Fisher Scientific), Waltham, Ma). Cells (1,000,000/mL) were cultured for 24 hours in 96-well flat-bottom plates in the presence of Influenza A/PR/8/34 (H1N1) 82 HA/ml (Charles River Laboratories, Wilmington, MA), PAM3 1 μg/ml and 10 μg/ml (Invivogen, San Diego, CA), 10 ng/mL GM-CSF, 0.1 μg/mL LPS (Invivogen, San Diego, CA), 100 μg/mL heat-killed *M*. *tuberculosis* (Invivogen), MOI 1 heat-killed *S*. *aureus* (Invivogen, San Diego, CA), and MOI 10 heat-killed *L*. *monocytogenes* (Invivogen, San Diego, CA). Blocking experiments were performed by pretreating pDCs 1 hour before stimulation with 1 μM CU-CPT22 (Merck-Millipore, Germany), Human TLR1 Neutralizing antibody—Monoclonal Mouse IgG1 (Invivogen, San Diego, CA), Human TLR2 Detection and Neutralizing antibody—Monoclonal Human IgA2 (Invivogen, San Diego, CA), Mouse IgG1 isotype control antibody (Invivogen, San Diego, CA), Human IgA2 Isotype Control (Invivogen, San Diego, CA). Supernatants were collected after 24-hours of stimulation and frozen until used.

### Cytokine quantification

Supernatants were collected after 24-hours of stimulation. Cytokine measurement was performed by Cytometric Bead Array Flex Set (BD Biosciences, Franklin Lakes, NJ). The following cytokines were measured in pDC supernatant: IL-6, IL-8, IFN-α, TNF-α, IP-10, and GZM-B, and for T cells: IL-2, IL-3, IL-4, IL-5, IL-6, IL-9, IL-10, IL-13, 1L-17A, IL-17F, GM-CSF, TNF-α, and IFN-γ. Acquisition was performed on a flow cytometer (BD LSR II), and data were analyzed using Fcap array (BD).

### Real time quantitative RT-PCR

Total RNA was extracted from freshly isolated, 1-hour PAM3-activated pDCs, freshly isolated CD11c^+^ DCs, and HeLa cells using RNeasy Micro kit (Qiagen, Netherlands) and processed as described by Volpe and colleagues [44]. The following probes (Life Technology (Thermo Fisher Scientific), Waltham, Ma) were used: TLR1 (Hs00413978_m1), TLR2 (Hs00152932_m1), TLR3 (Hs01551078_m1), TLR4 (Hs01060206_m1), TLR5 (Hs01019558_m1), TLR6 (Hs01039989_s1), TLR7 (Hs01933259_s1), TLR8 (Hs00152972_m1), TLR9 (Hs00370913_s1), TLR10 (Hs01935337_s1), B2M (Hs99999907_m1), GAPDH (Hs99999905_m1), and RPL34 (Hs00241560_m1). Crossing points (Cps) from each analyte were calculated using the second derivative maximum method, and the transcripts were quantified as fold changes in comparison to the mean of the 3 housekeeping genes (B2M, GAPDH, and RPL34).

### pDC–T cell cocultures

CD4^+^ naive T cells were stained with 5-(and 6)-Carboxyfluorescein diacetate succinimidyl ester (CFSE) (eBioscience (Thermo Fisher Scientific), Waltham, Ma). CD4^+^ naïve T cells were cultured for 6 days with allogeneic activated pDCs stimulated (FLU, gram+ bacteria treated, PAM3 in combination with anti-TLRs antibody), with CD11c+ DCs stimulated (FLU, PAM3) or with pDC DC5^−^ stimulated (LPS, FLU, PAM3, and 10 μg/mL GM-CSF) at a 5:1 ratio as previously described by Rissoan and colleagues [45]. CD4^+^ memory T cells were cultured for 6 days with allogeneic activated pDCs stimulated (FLU, gram+ bacteria treated, PAM3, and 10 μg/mL LPS, GM-CSF) at a 5:1 ratio as previously described by Rissoan and colleagues [45]. After coculture, T-cell expansion was determined by cell counting, and the percentage of dividing cells was determined by flow cytometer (BD LSR II). Supernatants were collected after 24 hours of polyclonal restimulation with anti-CD3/CD28 microbeads (LifeTech (Thermo Fisher Scientific), Waltham, Ma) and frozen until used.

### Statistical analysis

Statistical analyses were performed to compare the different conditions by Wilcoxon paired test using Prism (GraphPad Software, San Diego, Ca). Statistical significance was considered *p* < 0.05.

## Supporting information

S1 Fig pDCs from human tonsils respond to PAM3. Referring to Fig 1. (A) RT-PCR from total mRNA from sorted human pDCs. Results were normalized on 3 housekeeping genes. Results include 5 donors. (B) pDCs and CD11c^+^ DCs were stained in freshly isolated PBMCs with anti-TLR1 (left panel) and anti-TLR2 antibodies (right panel), respective cognate isotype. (C–D) Sorted human pDCs were cultured during 24-hours with medium (Ø), 0.1 μg/mL LPS, 1 and 10 μg/mL PAM3, 100 ng/mL GM, or 82 HA/ml FLU. (D) Surface expression of MHC-II complex from activated pDCs. Results include the mean of 9 donors. (E) Sorted tonsil pDCs were stimulated during 24 hours with only medium (Ø), 1 μg/mL PAM3, and 82 HA/ml FLU. Results include the mean of 4 donors. Surface expression of costimulatory or coinhibitory molecules from activated pDCs by FACS. *p < 0.05; **p < 0.01; ***p < 0.001 (Wilcoxon test). Underlying data for this figure can be found in S1 Data. CD, cluster of differentiation; FACS, fluorescence-activated cell sorting; FLU, influenza virus; GM, GM-CSF; GM-CSF, HA, hemagglutinin; granulocyte-macrophage colony-stimulating factor; LPS, lipopolysaccharide; PAM3, PAM3CSK4; pDC, Plasmacytoid predendritic cell; RT-PCR, real time PCR; TLR, toll-like receptor.
(TIF)

S2 Fig pDCs sense different gram+ bacteria through TLR1/2 pathway. Referring to Fig 2. (A–C) Sorted human pDCs were culture during 24 hours with only medium (Ø), DMSO, CU-CPT22, and FLU (in combination with DMSO and CU-CPT22). (A) Cell viability as percentage of cells DAPI negative. Results include the mean of 4 independent donors. (B) Surface expression of CD80 and CD86 from treated pDCs. Results include the mean of 4 independent donors. (C) Cytokine secretion by treated pDCs. Each dot represents an independent donor (*n* = 4). (D) Sorted human pDCs were cultured for 24 hours with only medium (Ø), heat-killed MT, heat-killed SA, heat-killed LM in the presence (+) or absence (−) of CU-CPT22. Surface expression of costimulatory molecules from activated pDCs. (E) The 24-hour stimulated pDCs and CD11c^+^ DCs (untreated, FLU, or 10 μg/mL PAM3) were cocultured with allogeneic CD4^+^ naive T cells for 6 days. Cytokines were measured after 24-hour polyclonal restimulation of the T cells. Results show 6 independent donors. Each dot represents a donor. *p < 0.05; **p < 0.01; ***p < 0.001 (Wilcoxon test). Underlying data for this figure can be found in S1 Data. CD, cluster of differentiation; CU-CPT22,; DC, dendritic cell; FLU, influenza virus; LM, *Listeria monocytogenes*; MT, *Mycobacterium tuberculosis*; PAM3, PAM3CSK4; pDC, Plasmacytoid predendritic cell; SA, *Staphylococcus aureus*; TLR, toll-like receptor.
(TIF)

S3 Fig PAM3-activated pDCs induce cytokine secrection from memory CD4^+^ T cells. Referring to Fig 2. (A) Memory CD4^+^ T cells were cultured with pDCs activated for 24 hours with only medium (NT), 100 ng/mL LPS, 1 μg/mL or 10 μg/mL PAM3, 10 ng/mL GM, or 82 HA/mL Influenza A/PR/8/34 (H1N1). Cytokines were measured in the supernatants by CBA after 6 days of coculture and 24 hours of restimulation with anti-CD3/CD28 beads. Mean ± SD from 6 independent donors. *p < 0.05 by paired Wilcoxon test. (B) Sort gating strategy of pure pDCs as LIN^−^CD4^+^CD11c^−^CD2^−^CD5^−^AXL^−^ (C) Quantification by CBA of cytokines produced by naive CD4 T cells cocultured with primary human pDCs activated for 24 hours with only medium (NT), 100 ng/mL LPS, 1 μg/mL or 10 μg/mL PAM3, 10 ng/mL GM, or 82 HA/mL Influenza A/PR/8/34 (H1N1). Cytokines were measured by CBA after 6 days of coculture and 24 hours of restimulation with anti-CD3/CD28 beads. Mean ± SD from 6 independent donors. *p < 0.05 by paired Wilcoxon test. Underlying data for this figure can be found in S1 Data. AXL, AXL receptor tyrosine kinase; CBA, cytokine bead array; CD, cluster of differentiation; FLU, influenza virus; GM, GM-CSF; LIN, lineage; LPS, lipopolysaccharide; NT, medium; PAM3, PAM3CSK4; pDC, Plasmacytoid predendritic cell.
(TIF)

S4 Fig TLR1/2 functional blocking differentially modifies CD4 T-cell activation. Referring to Fig 3. (A) Sorted human pDCs were cultured during 24 hours with only medium (Ø) and PAM3 in combination with TLR1 neutralizing antibody (αTLR1 Ab), TLR2 neutralizing antibody (αTLR2 Ab). Surface expression of costimulatory molecules from activated pDCs. (B–C) Allogeneic naïve CD4+ T-cell fold expansion and percentage of dividing cells after 6 days’ coculture with 24 hours PAM3 pDCs (in presence or absence of blocking antibodies). Results include the mean of 9 independent donors. Each dot is an individual donor. (D) Specific MFI of Th master regulator expression from PAM3 pDCs (in the presence or absence of neutralizing antibodies) T-cell coculture. Intracellular FACS was performed after 4 days of coculture. Results include the mean of 9 independent donors for Tbet, GATA3, and FOXP3. Results include the mean of 7 independent donors for BCL-6. (E) Th cytokine pattern from PAM3 (in combination with neutralizing antibody) activated T-cells coculture. Cytokines were measure after 24-hour polyclonal restimulation of the T cells. Results include the mean of 9 independent donors. *p < 0.05; **p < 0.01; ***p < 0.001 (Wilcoxon test). Underlying data for this figure can be found in S1 Data. Ab, anitbody; CD, cluster of differentiation; BCL-6, B-cell lymphoma 6; FACS, fluorescence-activated cell sorting; FOXP3, forkhead box P3; GATA3, GATA binding protein 3; MFI, mean fluorescence intensity; PAM3, PAM3CSK4; pDC, Plasmacytoid predendritric cell; Tbet, T-box transcription factor TBX21; Th, T helper; TLR, toll-like receptor.
(TIF)

S1 Data Numerical data used in this study. Numeric data shown in separate Excel spreadsheets (Microsoft, Redmond, WA).
(XLSX)

## Abbreviations

AKT: AKT serine/threonine kinase
AS-DC: AXL^+^SIGLEC6^+^
AU: arbitrary unit
AXL: AXL receptor tyrosine kinase
BCL-6: B-cell lymphoma 6
CD: cluster of differentiation
CFSE: carboxyfluorescein diacetate succinimidyl ester
Cp: crossing point
DAPI: 4,6-diamidino-2-phenylindole
DC: dendritic cell
DHX: DEAH box protein
FACS: fluorescence-activated cell sorting
FLU: influenza virus
FOXP3: forkhead box P3
GATA3: GATA binding protein 3
GM-CSF: granulocyte-macrophage colony-stimulating factor
GZMB: Granzyme B
ICOSL: inducible T cell costimulator ligand
IFN: interferon
IL: interleukin
IP-10: Interferon gamma-induced protein 10
IRF7: interferon regulatory factor 7
LPS: lipopolysaccharide
MAPK: Mitogen-activated protein kinases
NF-κB: nuclear factor kappa-light-chain-enhancer of activated B cells
PAM3: PAM3CSK4
PBMC: peripheral blood mononuclear cell
pDC: plasmacytoid predendritric cell
PDL1: programmed cell death ligand 1
PI3K: Phosphoinositide 3-kinase
TB: tuberculosis
Tbet: T-box transcription factor TBX21
Tfh: T-follicular helper
Th: T helper
TLR: toll-like receptor
TNF: tumor necrosis factor
Treg: T regulatory cell
