# Supplementary figures and images for: TLR1/2 orchestrate human plasmacytoid predendritic cell response to gram+ bacteria
Source: PLoS Biol. 2019 Apr 24;17(4):e3000209. doi: 10.1371/journal.pbio.3000209 (PMC6481764; doi:10.1371/journal.pbio.3000209)

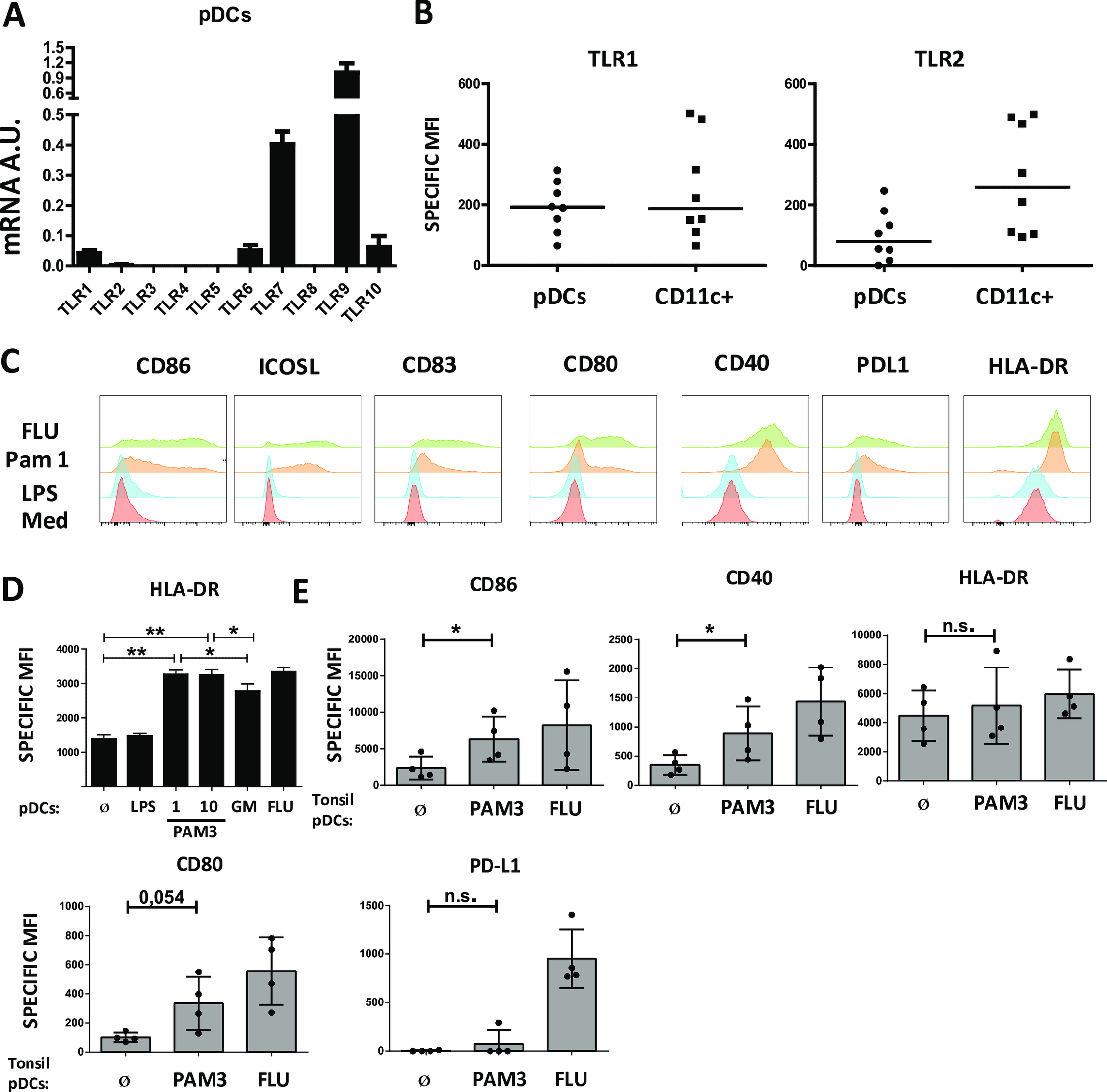

Supplement: S1 Fig — Referring to Fig 1. (A) RT-PCR from total mRNA from sorted human pDCs. Results were normalized on 3 housekeeping genes. Results include 5 donors. (B) pDCs and CD11c+ DCs were stained in freshly isolated PBMCs with anti-TLR1 (left panel) and anti-TLR2 antibodies (right panel), respective cognate isotype. (C–D) Sorted human pDCs were cultured during 24-hours with medium (Ø), 0.1 μg/mL LPS, 1 and 10 μg/mL PAM3, 100 ng/mL GM, or 82 HA/ml FLU. (D) Surface expression of MHC-II complex from activated pDCs. Results include the mean of 9 donors. (E) Sorted tonsil pDCs were stimulated during 24 hours with only medium (Ø), 1 μg/mL PAM3, and 82 HA/ml FLU. Results include the mean of 4 donors. Surface expression of costimulatory or coinhibitory molecules from activated pDCs by FACS. *p < 0.05; **p < 0.01; ***p < 0.001 (Wilcoxon test). Underlying data for this figure can be found in S1 Data. CD, cluster of differentiation; FACS, fluorescence-activated cell sorting; FLU, influenza virus; GM, GM-CSF; GM-CSF, HA, hemagglutinin; granulocyte-macrophage colony-stimulating factor; LPS, lipopolysaccharide; PAM3, PAM3CSK4; pDC, Plasmacytoid predendritic cell; RT-PCR, real time PCR; TLR, toll-like receptor. (TIF) [file pbio.3000209.s001.tif]

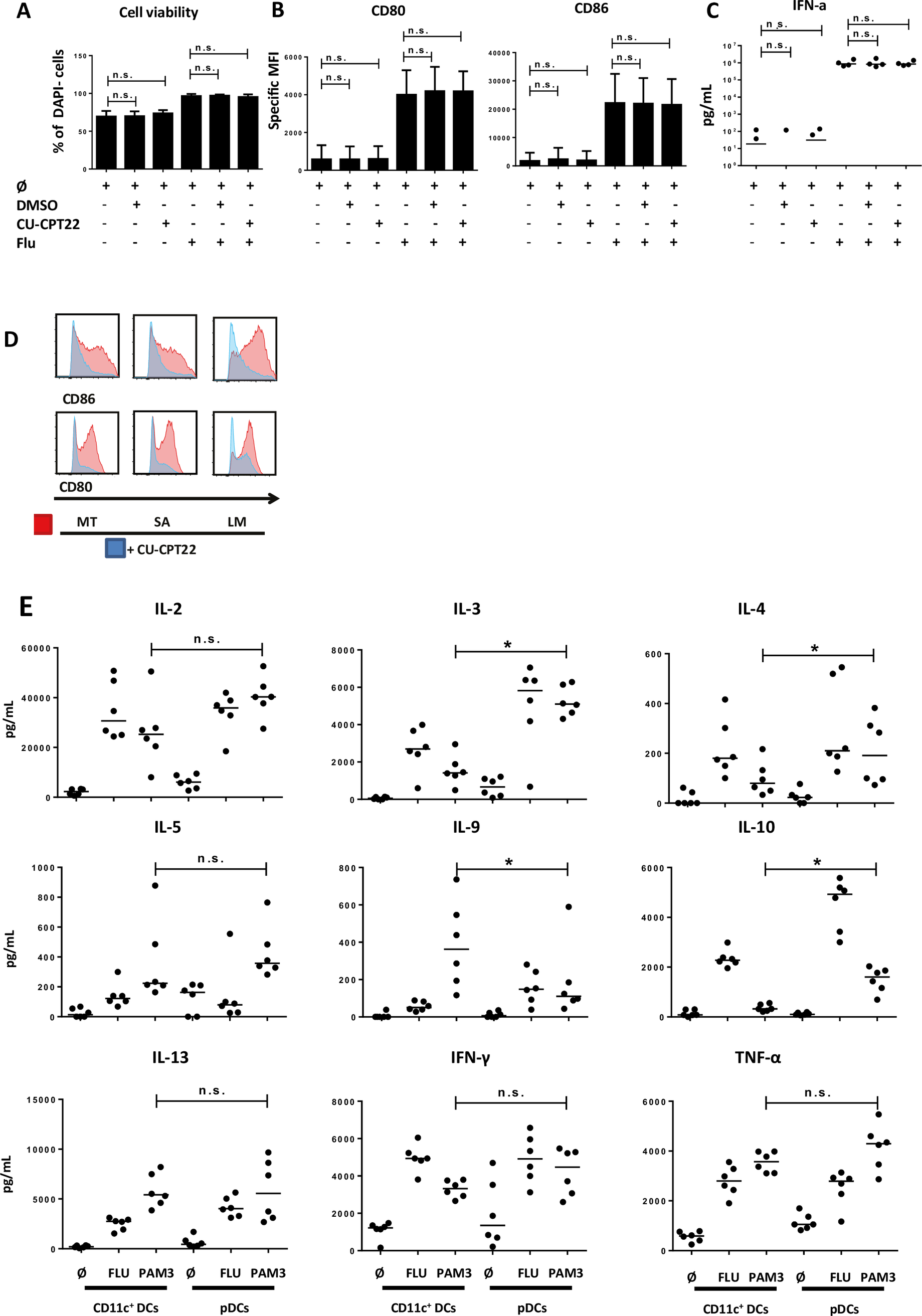

Supplement: S2 Fig — Referring to Fig 2. (A–C) Sorted human pDCs were culture during 24 hours with only medium (Ø), DMSO, CU-CPT22, and FLU (in combination with DMSO and CU-CPT22). (A) Cell viability as percentage of cells DAPI negative. Results include the mean of 4 independent donors. (B) Surface expression of CD80 and CD86 from treated pDCs. Results include the mean of 4 independent donors. (C) Cytokine secretion by treated pDCs. Each dot represents an independent donor (n = 4). (D) Sorted human pDCs were cultured for 24 hours with only medium (Ø), heat-killed MT, heat-killed SA, heat-killed LM in the presence (+) or absence (−) of CU-CPT22. Surface expression of costimulatory molecules from activated pDCs. (E) The 24-hour stimulated pDCs and CD11c+ DCs (untreated, FLU, or 10 μg/mL PAM3) were cocultured with allogeneic CD4+ naive T cells for 6 days. Cytokines were measured after 24-hour polyclonal restimulation of the T cells. Results show 6 independent donors. Each dot represents a donor. *p < 0.05; **p < 0.01; ***p < 0.001 (Wilcoxon test). Underlying data for this figure can be found in S1 Data. CD, cluster of differentiation; CU-CPT22,; DC, dendritic cell; FLU, influenza virus; LM, Listeria monocytogenes; MT, Mycobacterium tuberculosis; PAM3, PAM3CSK4; pDC, Plasmacytoid predendritic cell; SA, Staphylococcus aureus; TLR, toll-like receptor. (TIF) [file pbio.3000209.s002.tif]

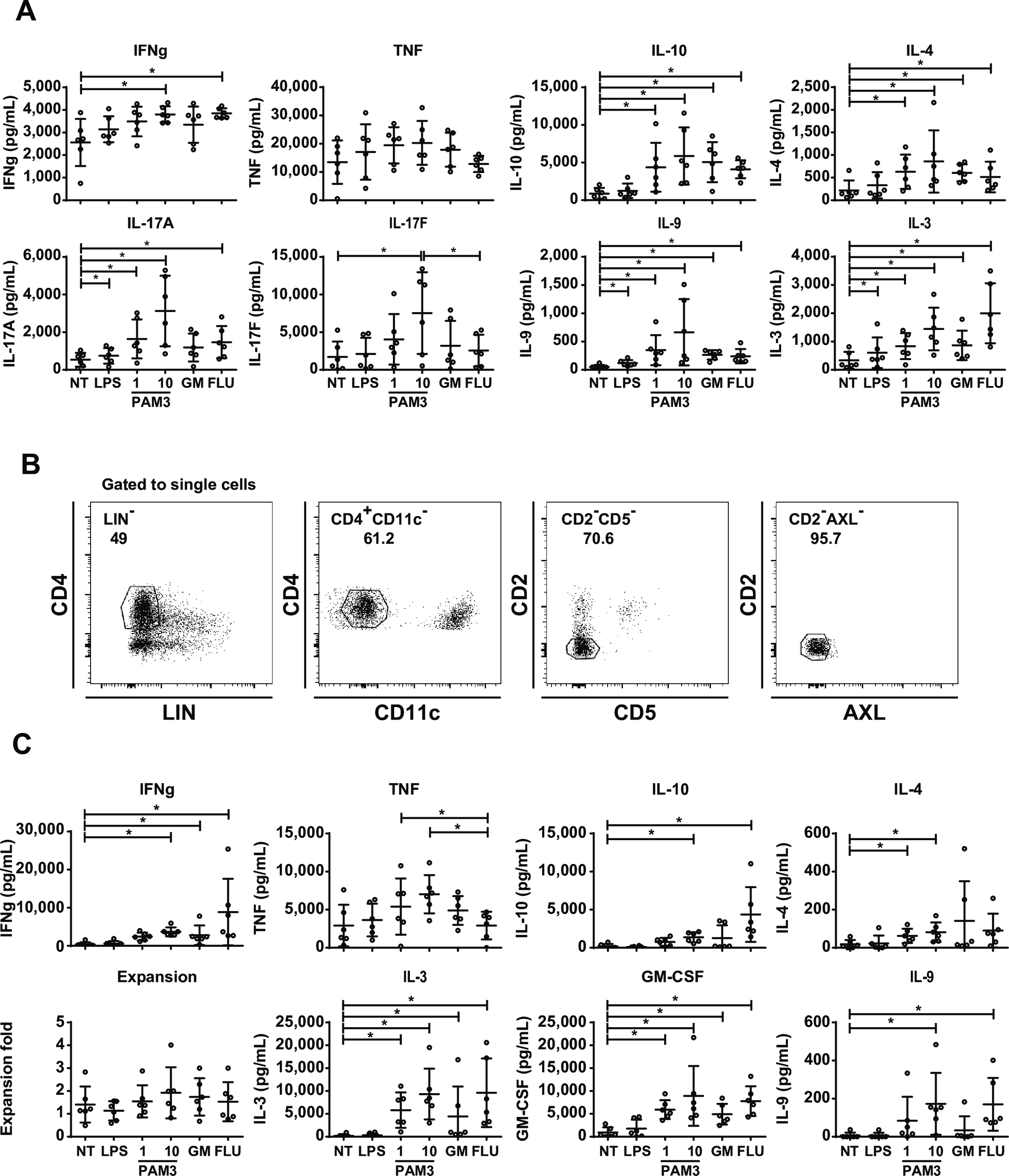

Supplement: S3 Fig — Referring to Fig 2. (A) Memory CD4+ T cells were cultured with pDCs activated for 24 hours with only medium (NT), 100 ng/mL LPS, 1 μg/mL or 10 μg/mL PAM3, 10 ng/mL GM, or 82 HA/mL Influenza A/PR/8/34 (H1N1). Cytokines were measured in the supernatants by CBA after 6 days of coculture and 24 hours of restimulation with anti-CD3/CD28 beads. Mean ± SD from 6 independent donors. *p < 0.05 by paired Wilcoxon test. (B) Sort gating strategy of pure pDCs as LIN−CD4+CD11c−CD2−CD5−AXL− (C) Quantification by CBA of cytokines produced by naive CD4 T cells cocultured with primary human pDCs activated for 24 hours with only medium (NT), 100 ng/mL LPS, 1 μg/mL or 10 μg/mL PAM3, 10 ng/mL GM, or 82 HA/mL Influenza A/PR/8/34 (H1N1). Cytokines were measured by CBA after 6 days of coculture and 24 hours of restimulation with anti-CD3/CD28 beads. Mean ± SD from 6 independent donors. *p < 0.05 by paired Wilcoxon test. Underlying data for this figure can be found in S1 Data. AXL, AXL receptor tyrosine kinase; CBA, cytokine bead array; CD, cluster of differentiation; FLU, influenza virus; GM, GM-CSF; LIN, lineage; LPS, lipopolysaccharide; NT, medium; PAM3, PAM3CSK4; pDC, Plasmacytoid predendritic cell. (TIF) [file pbio.3000209.s003.tif]

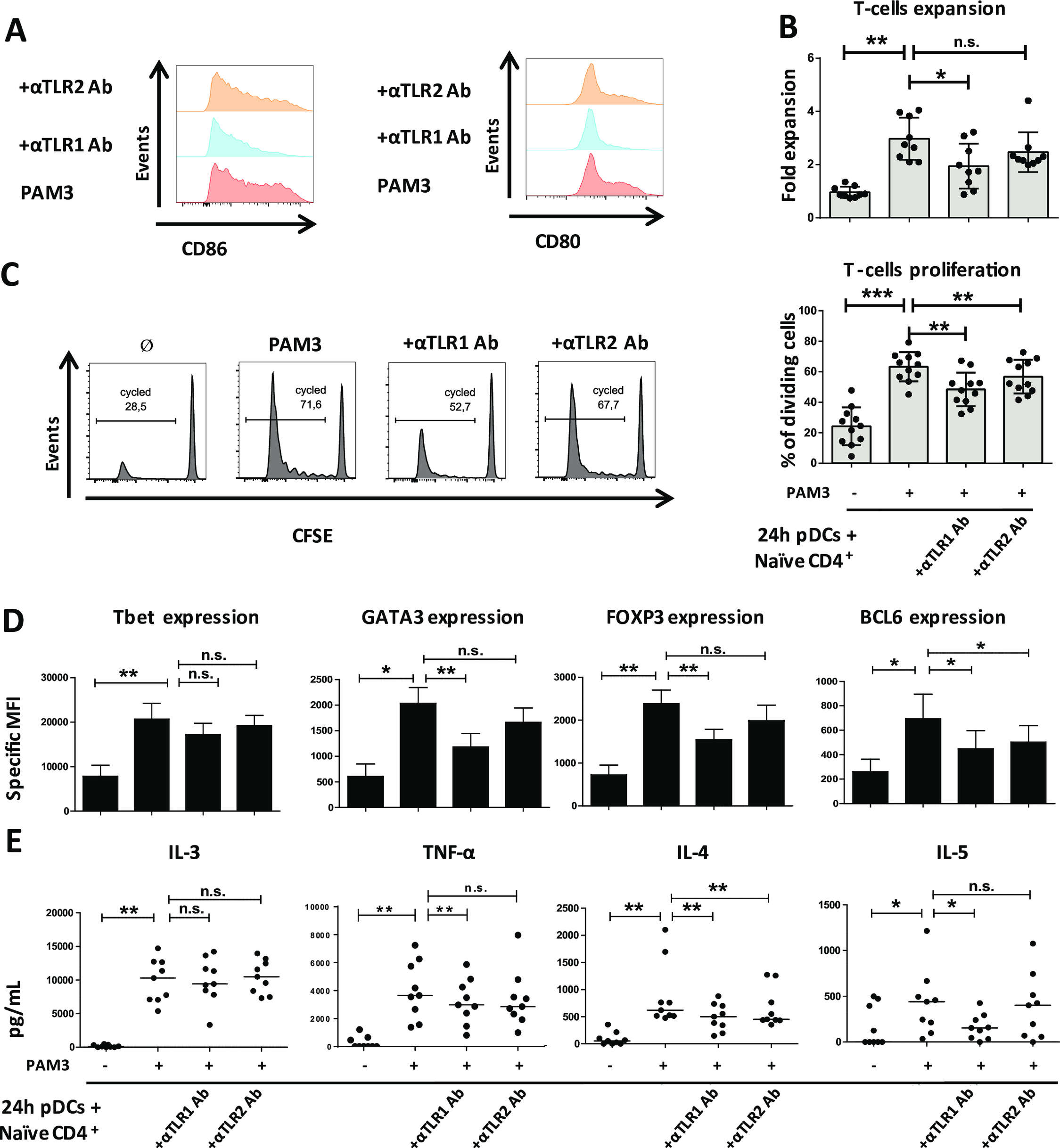

Supplement: S4 Fig — Referring to Fig 3. (A) Sorted human pDCs were cultured during 24 hours with only medium (Ø) and PAM3 in combination with TLR1 neutralizing antibody (αTLR1 Ab), TLR2 neutralizing antibody (αTLR2 Ab). Surface expression of costimulatory molecules from activated pDCs. (B–C) Allogeneic naïve CD4+ T-cell fold expansion and percentage of dividing cells after 6 days’ coculture with 24 hours PAM3 pDCs (in presence or absence of blocking antibodies). Results include the mean of 9 independent donors. Each dot is an individual donor. (D) Specific MFI of Th master regulator expression from PAM3 pDCs (in the presence or absence of neutralizing antibodies) T-cell coculture. Intracellular FACS was performed after 4 days of coculture. Results include the mean of 9 independent donors for Tbet, GATA3, and FOXP3. Results include the mean of 7 independent donors for BCL-6. (E) Th cytokine pattern from PAM3 (in combination with neutralizing antibody) activated T-cells coculture. Cytokines were measure after 24-hour polyclonal restimulation of the T cells. Results include the mean of 9 independent donors. *p < 0.05; **p < 0.01; ***p < 0.001 (Wilcoxon test). Underlying data for this figure can be found in S1 Data. Ab, anitbody; CD, cluster of differentiation; BCL-6, B-cell lymphoma 6; FACS, fluorescence-activated cell sorting; FOXP3, forkhead box P3; GATA3, GATA binding protein 3; MFI, mean fluorescence intensity; PAM3, PAM3CSK4; pDC, Plasmacytoid predendritric cell; Tbet, T-box transcription factor TBX21; Th, T helper; TLR, toll-like receptor. (TIF) [file pbio.3000209.s004.tif]
